# Seasonal Variation in the *β*‐Diversity of Periphytic Algae and Its Response to Landscape Patterns in the Chishui River, a Naturally Flowing Tributary of the Upper Yangtze River

**DOI:** 10.1002/ece3.70976

**Published:** 2025-02-06

**Authors:** Xiaopeng Tang, Haoyun He, Qiang Qin, Fei Xu, Fei Liu, Fubin Zhang

**Affiliations:** ^1^ College of Environmental Science and Engineering China West Normal University Nanchong China; ^2^ Institute of Hydrobiology Chinese Academy of Sciences Wuhan Hubei China

**Keywords:** biodiversity, Chishui River, periphytic algae, river ecosystems conservation

## Abstract

Understanding biodiversity is essential for preserving the stability of river ecosystems. However, the impact of landscape configurations and seasonal variations on biodiversity within undammed river ecosystems remains unexplored. Therefore, we selected the Chishui River—a naturally flowing tributary of the upper Yangtze River—for a survey of periphytic algae. The present study focuses on the seasonal fluctuations in the *β*‐diversity of periphytic algae within the Chishui River and its correlation with the surrounding landscape patterns. Our findings indicate that there is a substantial influence of seasonal variations on the community structure and *β*‐diversity of these algae within the Chishui River ecosystem. Concurrently, we observed that the turnover component predominantly contributes to *β*‐diversity. In light of these findings, we recommend that conservation measures be implemented across the entire Chishui River basin to safeguard the regional biodiversity. Redundancy analysis elucidated that water temperature, conductivity, and pH were the primary environmental drivers shaping the structure of periphytic algal communities. Furthermore, additional analyses using a random forest model indicated that landscape fragmentation and complexity were key determinants of *β*‐diversity in algal communities. Notably, the number of landscape patches was strongly correlated with the *β*‐diversity of periphytic algae. It is important to highlight that maintaining an optimal balance between the number of patches and their size is crucial for enhancing the ecosystem's capacity to preserve biodiversity. In summary, our findings provide insights into the interplay between biodiversity and land‐use practices within complex riverine environments, thereby offering a scientific foundation for the conservation and management of these ecosystems.

## Graphical abstract

Landscape fragmentation and complexity were key determinants of β‐diversity in algal communities. Effective biodiversity conservation requires comprehensive conservation efforts throughout the Chishui River basin. Ensuring a balanced distribution of habitat patch numbers and patch sizes is critical to maintaining the aquatic environment in the Chishui River basin.

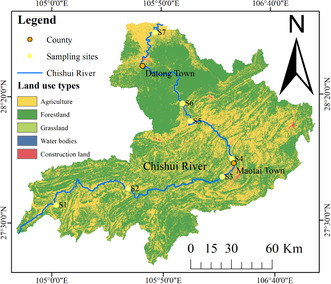

## 1 Introduction

Rivers play a central role in the global water cycle and are instrumental in providing essential services to ecosystems (Dalu et al. 2022; Ding et al. 2024). Within these ecosystems, biogeochemical cycling is significantly influenced by biodiversity (Fuß et al. 2024). Therefore, a comprehensive understanding of biodiversity distribution and the ecological forces that shape it is imperative for ensuring the vitality and proper functioning of riverine ecosystems (Zhu et al. 2023). Algae, as primary producers, substantially affect the structure and stability of ecosystems through variations in their community composition (Várbíró et al. 2020). Consequently, a thorough grasp of the spatiotemporal dynamics of algal communities in river systems is essential for biodiversity conservation and ecosystem management (Ma et al. 2024). However, algal community composition and diversity are shaped by complex interactions between biotic factors and environmental factors across various spatial scales (Oliveira et al. 2023). At the abiotic level, physical and chemical factors within river ecosystems display considerable spatial variability and temporal dynamics (Da Silva et al. 2024). These attributes lead to the observed spatiotemporal heterogeneity in the composition of algal communities (Huang et al. 2024). Furthermore, it is imperative to recognize that seasonal fluctuations significantly influence local environmental conditions (Liu, Zhang, et al. 2021). Consequently, temporal dynamics must be considered in algal ecological research to ensure a comprehensive understanding. Similarly, biological factors are also pivotal in influencing the structure of algal communities (Dalu et al. 2022). Within riverine ecosystems, top predators such as fish can modulate the abundance and diversity of primary producers through their interactions within the food web (Liu, Wang, et al. 2021). Moreover, land‐use patterns within watersheds significantly influence the structural composition of riverine biomes (Boakes et al. 2024). For instance, intensified land use can disrupt hydrological and chemical cycles within watersheds, leading to substantial consequences for biodiversity (Montràs‐Janer et al. 2024).

Given the profound impact of watershed land use on biodiversity, it is reasonable to infer that diverse land‐use patterns are instrumental in shaping the composition of algal communities (Han et al. 2024; Peng et al. 2021). In essence, modifications to the physical environment of streams, driven by shifts in land use, exert a substantial influence on the dynamics of algal assemblages (Falasco et al. 2021). For instance, regions with a higher percentage of soil exposure tend to exhibit increased nutrient runoff into water bodies (Akhtar et al. 2021). Consequently, this could significantly affect aquatic communities. However, varying intensities of land use generate diverse landscapes. This not only modifies the ecological functions of rivers but also has implications for species distribution (Fares et al. 2020; Paiva et al. 2021). Rivers are inherently positioned as sentinels for the effects of landscape change, due to their unique role at the lowest elevations within the topographic level (Ren et al. 2020). Likewise, distinct land‐use types and intensities within a watershed can exert specific influences on aquatic organisms across various temporal and spatial scales (Mello et al. 2023). Furthermore, alterations in land use have escalated surface runoff. Such alterations modify the sediment texture of the streambed and the hydrodynamic properties of water flow (Granitto, Lopez, and Rodríguez 2022). Consequently, a comprehensive investigation into the interplay between local environmental factors, surrounding landscape characteristics, and algal communities is crucial for understanding the precise effects of human‐induced activities on the biodiversity of river ecosystems (Da Silva et al. 2024).

At the spatial scale, biodiversity is typically categorized into three fundamental dimensions: *α*‐diversity, *β*‐diversity, and *γ*‐diversity (Socolar et al. 2016). By examining *α*‐diversity and *γ*‐diversity, researchers can employ statistical methods to quantitatively assess the species abundance dynamics within and between different communities (Dunck, Felisberto, and Nogueira 2019). In contrast to *α*‐diversity and *γ*‐diversity, *β*‐diversity specifically highlights the variability in community composition across spatial and temporal gradients (Chen et al. 2020). *β*‐diversity is recognized as a critical indicator of community stability (Falasco et al. 2021). Therefore, it has gained increasing emphasis in ecological research (Leboucher et al. 2019). As our understanding of species diversity within communities has progressed, *β*‐diversity was further differentiated into turnover and nestedness components (Baselga 2010). High turnover rates imply a significant rate of species interchange between different locations, while high nestedness points to considerable variation in species across points (Baselga 2010). This distinction is instrumental in uncovering the ecological processes that influence algal communities (Wu et al. 2021). Consequently, *β*‐diversity is pivotal for pinpointing the forces that shape community composition and the spatial distribution of biodiversity.

Empirical studies have shown that landscape pattern changes have profound effects on biodiversity (Martínez‐Núez, Martínez‐Prentice, and García‐Navas 2023; Newbold et al. 2015). For instance, algal communities may exhibit adaptive responses to land‐use alterations by modulating *β*‐diversity (Bomfim et al. 2023). Prior research has suggested that taxonomic *β*‐diversity within algal communities is primarily driven by species turnover, while functional *β*‐diversity is predominantly shaped by nestedness (Wu et al. 2021). Accordingly, Within the framework of assessing how land‐use patterns shape community structure, *β*‐diversity serves as a critical indicator. It enables the identification of areas that require targeted conservation efforts (Heino, Melo, and Bini 2015). This underscores the importance of integrating *β*‐diversity analysis into the development of biodiversity protection strategies.

Periphytic algae serve as ideal model organisms for studying the effects of environmental drivers and landscape pattern changes on community composition (Han et al. 2024; Oliveira et al. 2023). In recent years, they have been widely employed as bioindicators to assess the consequences of environmental shifts and intensifying land use on aquatic health (Gao et al. 2024; Lee et al. 2021; Peng et al. 2020). These organisms play pivotal roles in the functioning of river ecosystems, actively participating in the biogeochemical cycling of nutrients and energy flow (Sofi et al. 2023). Compared to other biomes, periphytic algae exhibit shorter life cycles and a higher sensitivity to environmental perturbations (Saxena et al. 2021). Consequently, they serve as reliable bioindicators for assessing the integrity of aquatic environments (Zorzal‐Almeida and Fernandes 2021). Although a robust correlation exists between algal diversity and the dynamics of environmental change and land use, the underlying mechanisms of this association remain incompletely understood and require further elucidation.

Within this research framework, our objective is to conduct a comprehensive analysis of the relationship between periphytic algal diversity and environmental change and land‐use factors, with the goal of establishing effective ecological assessment indicators. To this end, the study is focused on the Chishui River, a naturally flowing tributary of the upper Yangtze River. The upper Yangtze River basin is known for its diverse aquatic fauna and is particularly noted for its high number of endemic species (Liu, Wang, et al. 2021). However, over the past few decades, numerous species have faced the threat of extinction or have been classified as critically endangered due to escalating human activities (Yu et al. 2022). The Chishui River basin possesses a highly heterogeneous natural environment, characterized by a variable topography and a variety of landscape types (Zhang, Cai, and Tu 2023). Such characteristics render the Chishui River an optimal site for examining the diversity of periphytic algae across diverse land‐use patterns. The objectives of our study were to (1) quantify the seasonal variations in the composition and *β*‐diversity of periphytic algal communities, (2) identify key ecological drivers of community dynamics by analyzing the relationship between environmental variables and biota abundance, (3) integrate landscape pattern indices with biodiversity metrics to pinpoint areas that significantly contribute to biodiversity, thereby informing conservation prioritization and planning strategies. Our research endeavors to deepen the comprehension of how riverine periphytic algae biodiversity is influenced by fluctuating environmental parameters and land‐use practices. The aim is to forecast algal responses to these changes and establish a scientific baseline for the conservation of aquatic environments.

## 2 Materials and Methods

### 2.1 Study Area

**FIGURE 1:**
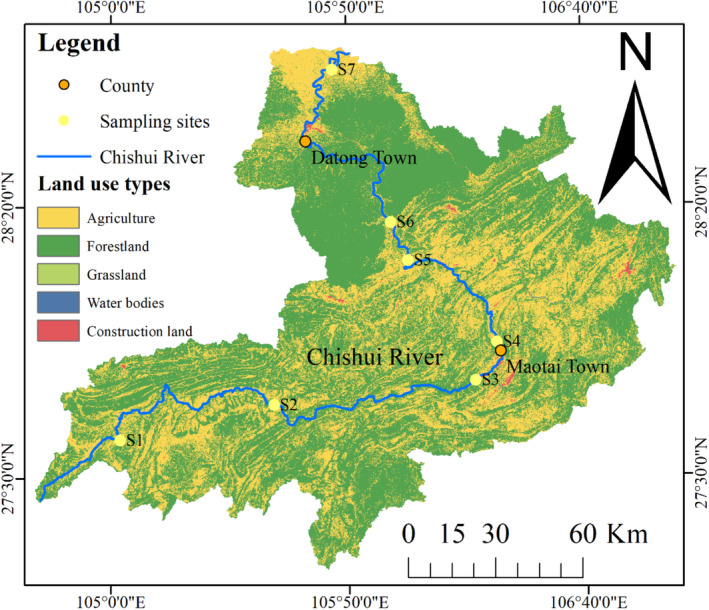
Sampling sites and land use types in the Chishui River basin.

This research is centered on the Chishui River (Figure 1), situated in the southwestern region of China. It serves as a significant tributary to the upper reaches of the Yangtze River (Liu, Wang, et al. 2021). The basin covers an area of 20,440 km^2^ and the river extends for 436.5 km (Wang et al. 2019). The Chishui River is a critical biodiversity hotspot, attributed to the absence of cascade hydropower stations, which allows it to retain a more pristine ecological condition (Yu et al. 2022). Characterized by a monsoon climate, the basin experiences an average rainfall of 892.4 mm, primarily in the summer. Consequently, the Chishui River is prone to seasonal flooding, which can exert substantial influences on the aquatic ecosystem (Liu, Zhang, et al. 2021).

The Chishui River originates in Chishuiyuan Town, Zhenxiong County, Yunnan Province, China and traverses through Hejiang County, Sichuan Province, before ultimately discharging into the Yangtze River. This river is strategically positioned at the tri‐junction of Yunnan, Sichuan, and Guizhou Provinces in the southwestern region of China (Wu and Wang 2024). This watershed's primary land‐use types comprise farmland and forestland, contributing to the area's distinctive natural landscape (Zhu et al. 2024). The Chishui River basin is divided into upper, middle, and lower regions based on its topographical variations, elevation gradients, and hydrological characteristics, with the demarcation at the towns of Maotai and Datong. The upper and middle sections are characterized by deep valleys and rapid currents, whereas the lower reaches are marked by broader river channels and slower flow velocities.

### 2.2 Sampling and Processing

Sampling of periphytic algae in the Chishui River was conducted during the winter of 2020 (December), spring of 2021 (April), and summer of 2021 (August). Five cobbles were randomly selected within a 100‐m stretch both upstream and downstream of the sampled reach. For each stone, a flat surface area of 9 cm^2^ was identified using a 30 mm × 30 mm^2^ piece of plastic. Subsequently, a hard‐bristled toothbrush was employed to collect samples of periphytic algae. The toothbrush and pebbles were first cleaned with distilled water to ensure sample integrity. Following collection, the samples were consolidated into a single composite for analysis. They were immediately preserved in 50 mL sample bottles, with the inclusion of acetic Lugol solution to maintain their viability. Upon arrival at the laboratory, the samples underwent meticulous examination under a microscope, with a focus on classifying the algal specimens to the detailed taxonomic level possible (genus or species). This identification process is facilitated by reference to specialized literature (Hu and Wei 2006).

### 2.3 Environmental Characteristics

We utilized a multi‐parameter water quality analyzer (YSI Pro Plus, YSI Inc., USA) to assess several key water quality parameters. This instrument provided precise measurements of water temperature (WT), dissolved oxygen (DO) levels, electrical conductivity (Cond), and pH, offering a comprehensive analysis of the aquatic environment. Additionally, a portable velocity analyzer (LS‐300A, China) was utilized to determine stream flow velocity (Vel). In each sampling reach, triplicate measurements of key environmental indicators were conducted and the averages were calculated to represent the environmental parameters at each site for subsequent analyses. Additionally, at each sampling location, we collected composite water samples, which were promptly placed in a portable refrigeration unit to preserve their integrity throughout transportation to the laboratory. In the laboratory, a comprehensive analysis was conducted on these samples to evaluate a range of water quality parameters, specifically focusing on total nitrogen (TN) and total phosphorus (TP) concentrations.

Data on water levels and flows for the Chishui River were obtained from the National Water and Rainfall Information Network (http://xxfb.mwr.cn/sq_djdh.html). For our analysis, land‐use data encompassing the study region were extracted from the China Land Cover Data set (CLCD) (Yang and Huang 2021). These data were subsequently processed using the ArcGIS 10.7 software suite. We consolidated the initial nine detailed land‐use classifications into a more manageable framework of five primary categories: agricultural land, forestland, grassland, water bodies, and construction land (Zhang et al. 2024).

### 2.4 Diversity Indices

This study employs the *β*‐diversity decomposition method proposed by Baselga (Baselga 2010), employing the Sørensen dissimilarity index to measure the various facets of *β*‐diversity within our study region. This includes the total *β*‐diversity (*β*_sor_), the turnover component (*β*_sim_), and the nestedness component (*β*_sne_). To elucidate the proportional impact of the turnover component on the total *β*‐diversity (*β*_ratio_ = *β*_sim_/*β*_sor_), we calculated the *β*_ratio_. A *β*_ratio_ value exceeding 0.5 suggests that species turnover predominantly drives *β*‐diversity, whereas a *β*_ratio_ value below 0.5 indicates that species nestedness is the primary determinant of *β*‐diversity.

In this study, landscape fragmentation was quantified using three primary metrics: number of patches (NP), total edge (TE), and edge density (ED). To evaluate landscape heterogeneity, we employed the Shannon diversity index (SHDI) and the Shannon evenness index (SHEI). Furthermore, landscape complexity was analyzed employing the landscape shape index (LSI), the largest patch index (LPI), and the interspersion juxtaposition index (IJI). These indices enable a comprehensive analysis of land use patterns across multiple dimensions, including composition, morphology, and distribution, thereby revealing the spatial heterogeneity of the surface landscape (Zhang et al. 2022). The computation of these landscape indices was facilitated by the FRAGSTATS 4.2 software.

### 2.5 Data Analysis

In our investigation, we utilized one‐way analysis of variance (ANOVA) to evaluate significant seasonal variations in environmental parameters and the composition of periphytic algal communities. To quantify species diversity, we deployed the Shannon‐Wiener index, the Simpson index, and the Pielou evenness index. These indices were computed using the “vegan” package (Oksanen et al. 2024). Furthermore, the species presence–absence matrix was employed to calculate *β*‐diversity and its components. The calculation of *β*‐diversity was computed using the “betapart” package (Baselga et al. 2023).

Additionally, to determine the influence of seasonal fluctuations on the *β*‐diversity of the periphytic algae community, this study employed principal coordinate analysis (PCoA) and analysis of similarities (ANOSIM) based on a similarity matrix. Moreover, to evaluate the correlation between periphytic algal abundance and environmental factors, we applied redundancy analysis (RDA). The choice of RDA was informed by the gradient length, which was found to be shorter than three standard units, suggesting a linear relationship between species distribution and environmental gradients. Before conducting redundancy analysis (RDA), we screened for multicollinearity among environmental drivers using variance inflation factors (VIF). All statistical analyses were performed utilizing the “vegan” package within the R programming environment (Oksanen et al. 2024).

To assess the influence of landscape pattern indices on the *β*‐diversity of periphytic algal communities, we initially utilized Spearman's rank correlation coefficient to explore the association between these variables. Subsequently, a random forest model was developed to quantify the specific influence of individual landscape pattern indices on *β*‐diversity. The model development was facilitated utilizing the “randomForest” package within the R statistical software environment (Liaw and Wiener 2002).

## 3 Result

### 3.1 Hydrological Changes

**FIGURE 2:**
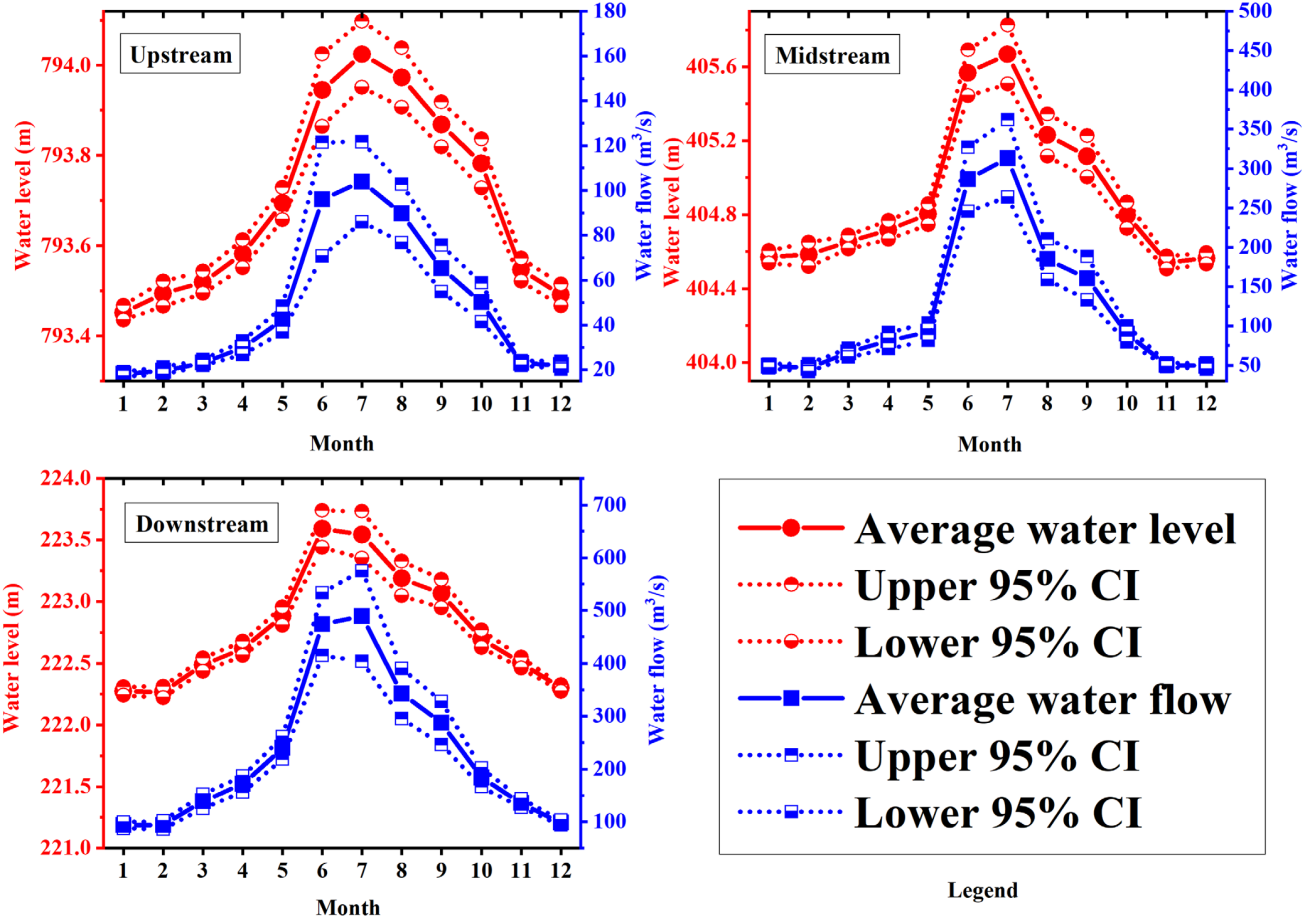
Hydrological changes of the upstream, midstream, and downstream of the Chishui River.

The Chishui River basin, influenced by the monsoon climate, exhibits three distinct hydrological seasons. Hydrological data analysis from 2013 to 2020 (Figure 2) revealed that December, corresponding to winter, represents the dry season, whereas August, during summer, is the wet season. April, in the spring, marks the normal season with moderate hydrological activity.

### 3.2 Environmental Variables

In our research, we have compiled environmental variable data for the study region (Table 1). This study area is characterized by elevated dissolved oxygen levels and reduced flow velocities. There exists a considerable variation in conductivity and dissolved oxygen values. During the wet season, water temperatures were significantly higher compared to those observed during the dry and normal seasons. The average dissolved oxygen levels in the study area were higher during the dry and normal seasons (8.12 mg/L and 8.78 mg/L, respectively). In contrast, the wet season exhibited significantly lower averages, with a mean concentration of 6.04 mg/L. Furthermore, mean conductivity values exhibited a greater than 50% reduction during the rainy season relative to those recorded in other seasons. In the dry season, pH levels and total phosphorus concentrations were at their lowest, with a subsequent increase observed during both the wet and normal seasons. The average flow velocity peaked at 0.49 m/s in the wet season, subsequently dropping to 0.27 m/s in the dry season. Total nitrogen concentrations peaked in the dry season and were comparatively lower in the wet season. Notably, one‐way ANOVA revealed significant differences in water temperature, conductivity, and pH among seasons (*p* < 0.05).

**TABLE 1:** Seasonal variations in environmental parameters in the Chishui River basin.

	Wet season	Normal season	Dry season	*F*	*p*
WT (°C)	23.1 ± 2.7	18.6 ± 3.5	13.3 ± 1.2	23.419	< **0.001**
DO (mg/L)	6.04 ± 0.82	8.12 ± 4.87	8.78 ± 1.67	1.571	0.235
Cond (s/cm)	212.3 ± 146.3	503.6 ± 92.0	458.1 ± 81.1	14.147	< **0.001**
pH	8.21 ± 0.31	8.81 ± 0.58	7.77 ± 0.85	4.963	**0.019**
Vel (m/s)	0.49 ± 0.35	0.31 ± 0.21	0.27 ± 0.26	1.316	0.293
TN (mg/L)	0.98 ± 0.60	1.35 ± 0.59	1.42 ± 0.34	1.396	0.273
TP (mg/L)	0.10 ± 0.05	0.08 ± 0.10	0.04 ± 0.05	1.058	0.368

*Note:* The bolded portions mean significant variations.

Abbreviations: Cond, conductivity; DO, dissolved oxygen; TN, total nitrogen; TP, total phosphorus; Vel, velocity; WT, water temperature.

### 3.3 Community Structure of Periphytic Algae

**FIGURE 3:**
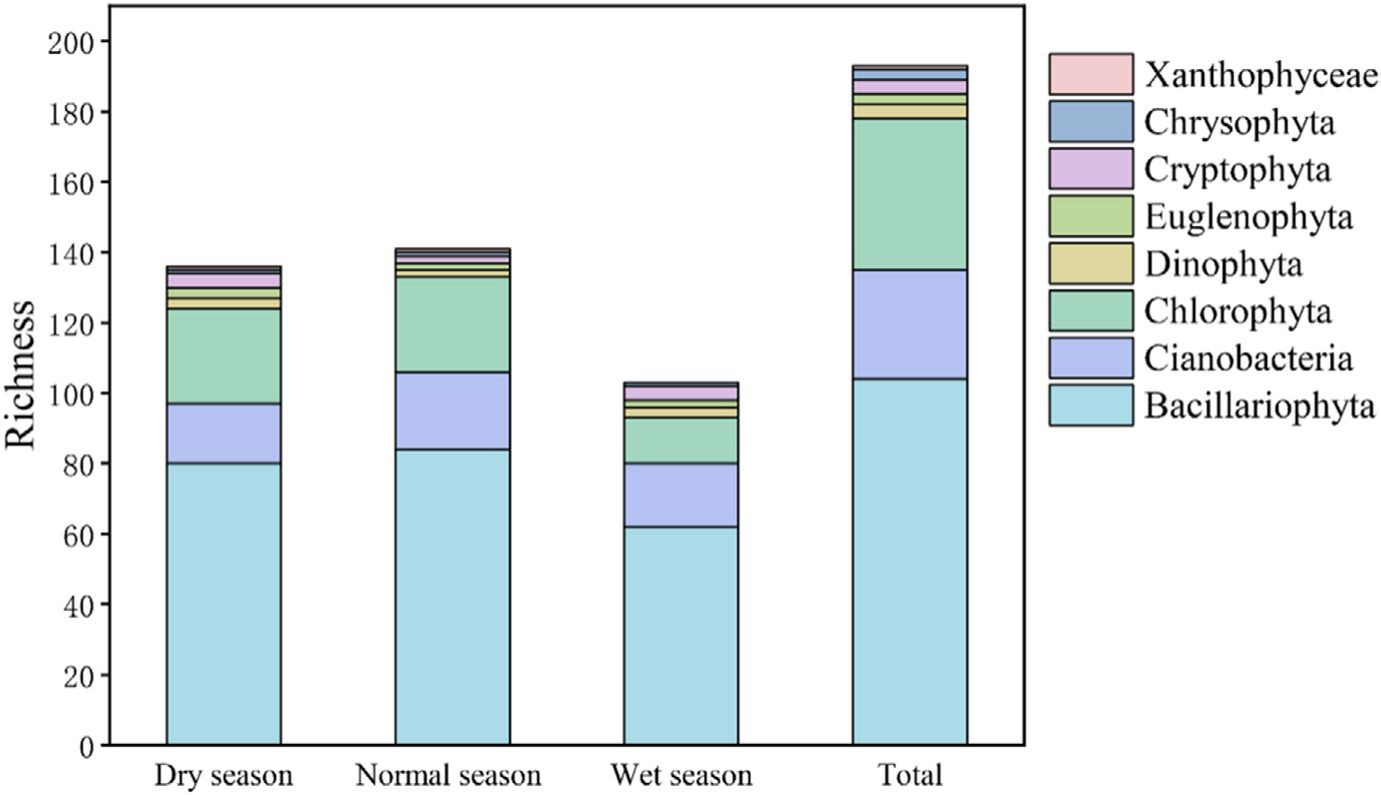
Species richness of periphytic algae in the Chishui River.

In this study, we identified 193 taxonomic units of periphytic algae across 8 phyla and 85 genera. The Bacillariophyta was predominant, comprising 104 species (53.9% of the total). The Pennate was the most dominant component within the Bacillariophyta. It was found to comprise 94 species distributed across 23 genera, 8 families, and 5 orders. The density of Pennate was 7.59 × 10^5^ ind./cm^2^, which accounted for 91.90% of the total diatom density. It was followed by Chlorophyta with 43 species and Cyanobacteria with 31 species (Figure 3). Both the Dinophyta and Cryptophyta contained four species each. The Euglenophyta and Chrysophyta each harbored three species, whereas the Xanthophyceae were represented by a single species. Analysis of species distribution among periphytic algae (Table 2) revealed significant seasonal variations (*p* < 0.05). Bacillariophyta density was markedly higher during the normal and dry seasons relative to the wet season. Conversely, the relative abundance of Cyanobacteria peaked during the wet season. Furthermore, Bacillariophyta constituted over 80% of the total density in both dry and normal seasons, representing the dominant component of the periphytic algae community. It is particularly noteworthy that the density of periphytic algae exhibited a significant decrease in the wet seasons relative to those recorded in other seasons.

**TABLE 2:** Relative abundance of various species of periphytic algae in the Chishui River.

	Wet season	Normal season	Dry season	*F*	*p*
Abundance (ind./cm^2^)	3785.7 ± 1306.7	151,980.0 ± 62,150.6	272,078 ± 87,376.5	32.984	< **0.001**
Relative abundance (%)					
Bacillariophyta	53.3 ± 19.6	80.6 ± 20.2	81.5 ± 7.1	6.422	**0.008**
Cyanobacteria	35.6 ± 23.9	8.3 ± 5.2	10.8 ± 5.4	7.601	**0.004**
Chlorophyta	6.6 ± 4.7	10.4 ± 15.0	6.3 ± 3.2	0.432	0.656
Dinophyta	2.1 ± 4.7	0.1 ± 0.2	0.5 ± 0.4	1.091	0.357
Euglenophyta	0.3 ± 0.4	0.1 ± 0.2	0.2 ± 0.2	0.231	0.796
Cryptophyta	1.2 ± 1.2	0.3 ± 0.5	0.6 ± 0.9	2.127	0.148
Chrysophyta	0.9 ± 2.4	0	0	0.925	0.415
Xanthophyceae	0	0.1 ± 0.1	0.1 ± 0.1	1.545	0.240

*Note:* The bolded portions mean significant variations.

### 3.4 Seasonal Variations in the Diversity of Periphytic Algae Community

**FIGURE 4:**
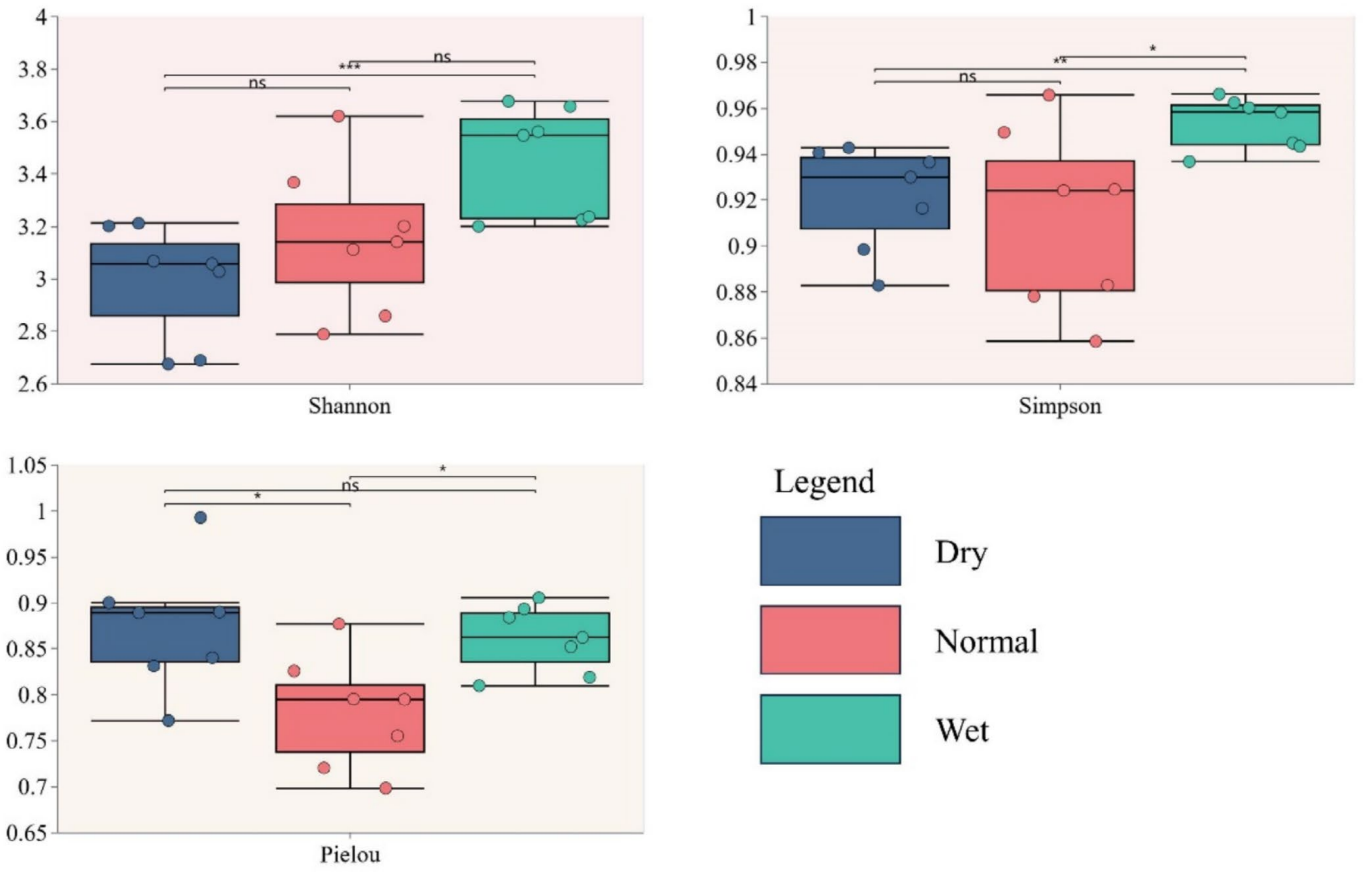
Seasonal variations in the species diversity of periphytic algae. ns, not significant, *p < 0.05; **p < 0.01, ***p < 0.005.

Seasonal fluctuations significantly influenced the species diversity of the periphytic algal communities (Figure 4). The Shannon‐Wiener and Simpson diversity indices demonstrated a pronounced increase in the dry season, whereas the Pielou evenness index exhibited a significant decline during the normal season. It is noteworthy that the Shannon‐Wiener and Simpson indices revealed significant differences between the rainy and dry seasons, whereas the Pielou index did not reveal significant seasonal variation between these two periods.

**FIGURE 5:**
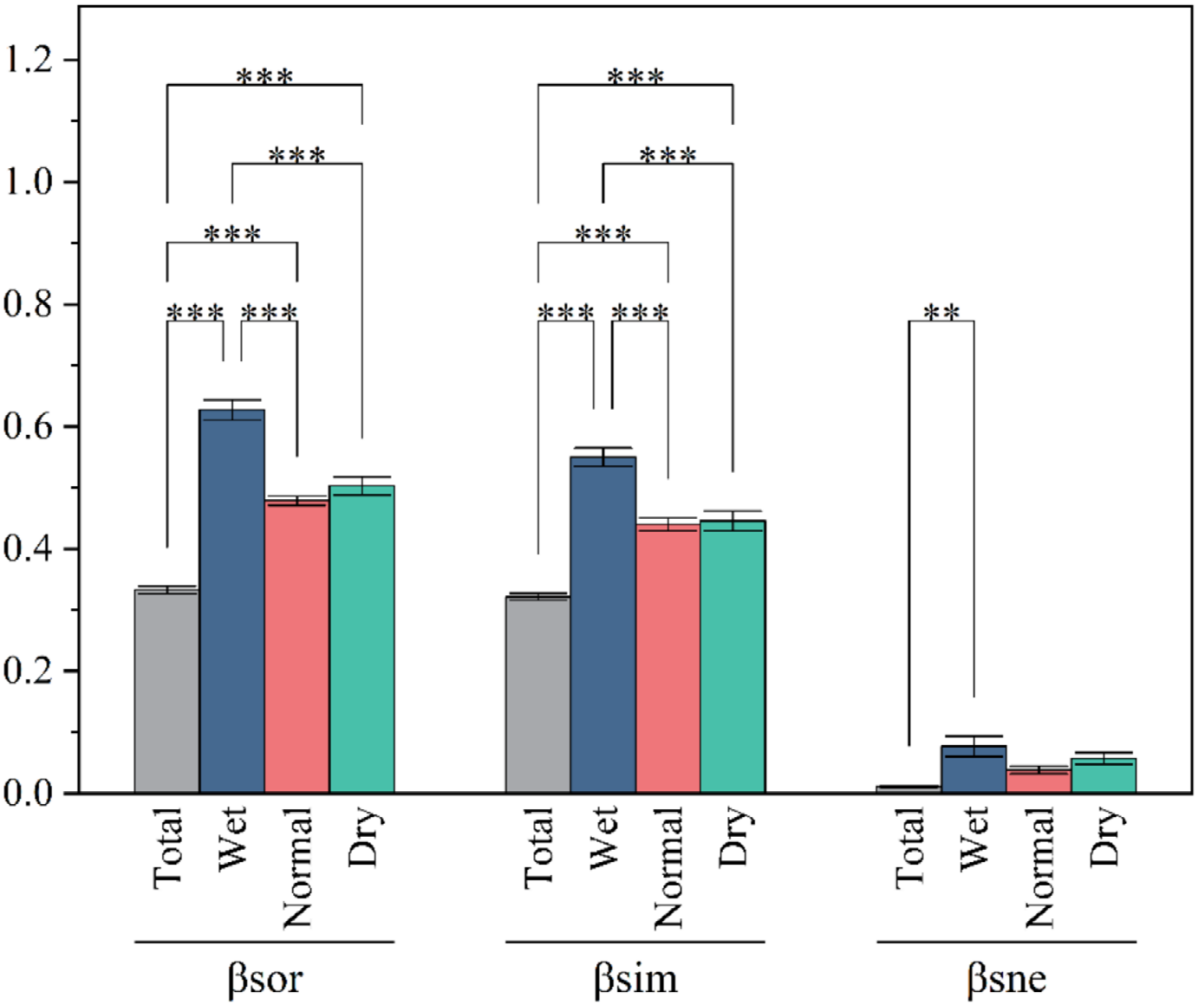
Seasonal variations in *β*‐diversity and its components of periphytic algae. total, total of three seasons. ** *p* < 0.01, ***p < 0.005.

The periphytic algal community within our study area showed mean values for *β*‐diversity and its components (turnover and nestedness) of 0.333, 0.322, and 0.011, respectively (Figure 5). Given that the *β*_ratio_ exceeded 0.5, this indicates that the turnover component was the primary driver of *β*‐diversity in this region.

**FIGURE 6:**
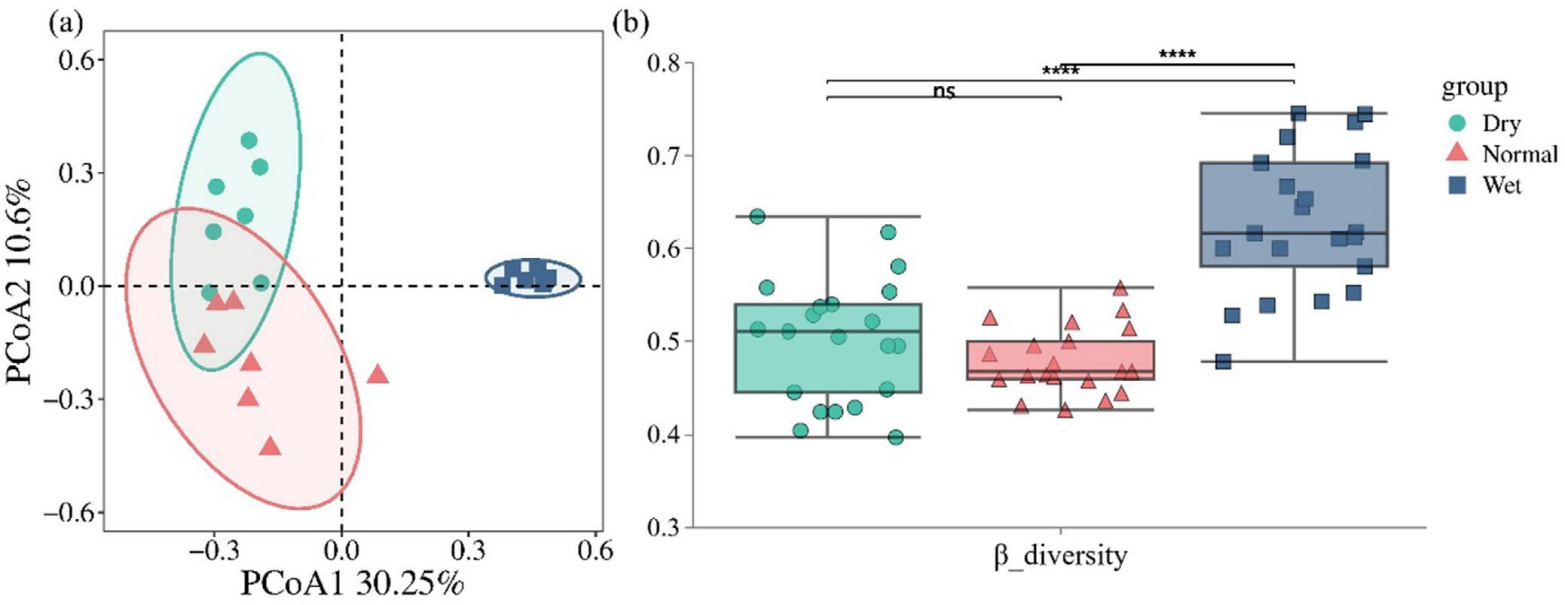
(a) Principal coordinates analysis of seasonal variations in periphytic algae. (b) Boxplot showing the *β*‐diversity of the periphytic algae community in different seasons. ns, not significant, ****p < 0.001. *β*_diversity, *β*‐diversity.

**FIGURE 7:**
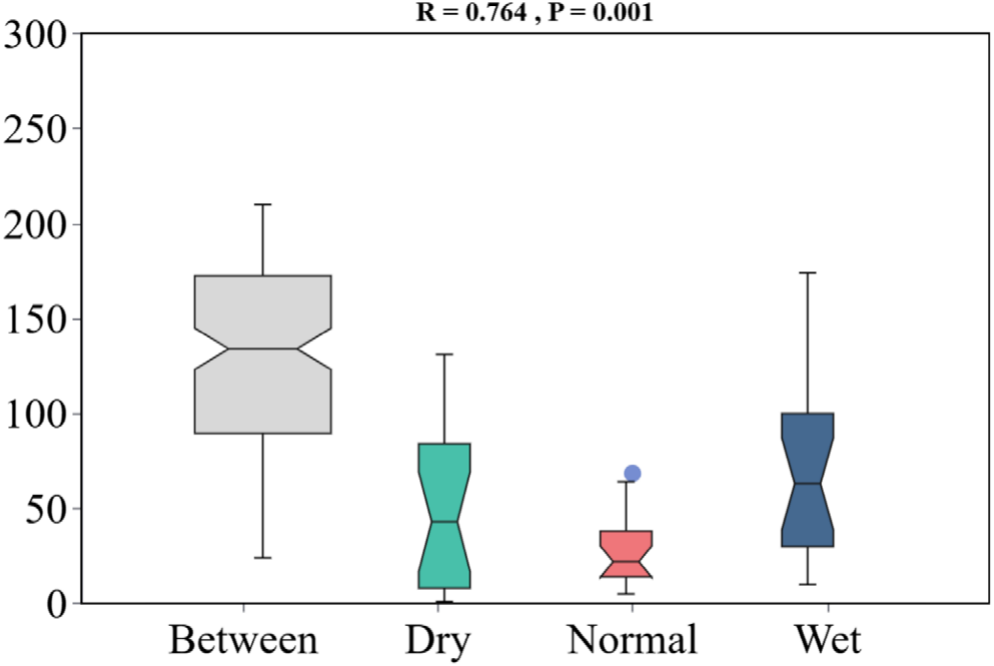
Results of similarity analyses in different seasons of periphytic algae communities.

To assess *β*‐diversity, we performed a principal coordinate analysis (PCoA) to explore the differences in the composition of periphytic algal communities throughout the seasons. The PCoA results highlighted significant seasonal variations within the algal communities, explaining approximately 40.85% of the total variance (Figure 6a). Moreover, the boxplot (Figure 6b) indicated that *β*‐diversity was highest in the wet season, subsequently declining through the dry season, reaching its minimum in the normal season. The outcomes of the analysis of similarities (ANOSIM), as depicted in Figure 7, substantiated the presence of statistically significant differences in algal community composition across seasons (*R* = 0.764, *p* = 0.001).

### 3.5 Redundancy Analysis (RDA) of Periphytic Algae Communities With Environmental Variables

**FIGURE 8:**
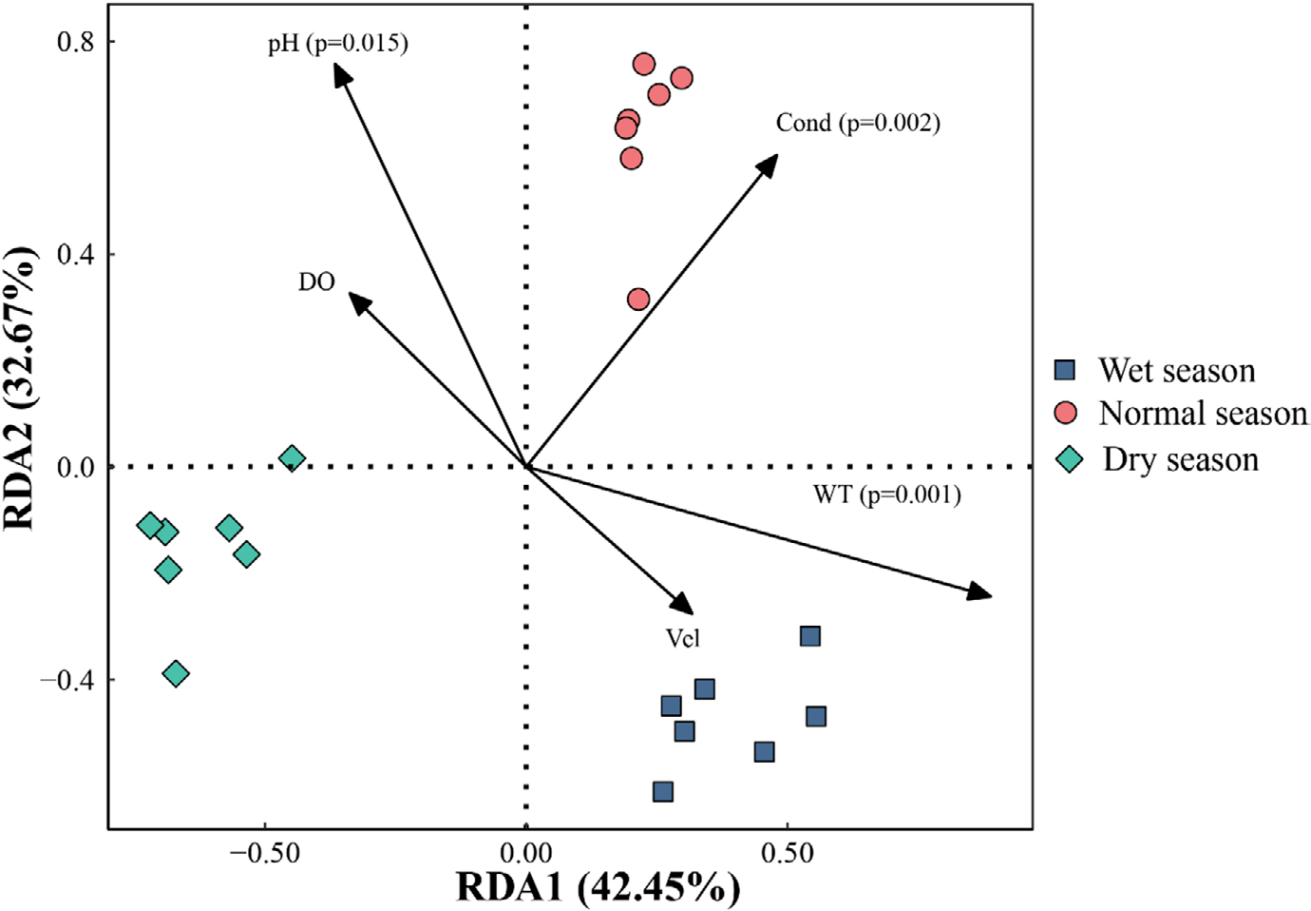
RDA of periphytic algae communities with environmental variables. Cond, electrical conductivity; DO, dissolved oxygen; Vel, flow velocity; WT, water temperature.

Before conducting RDA, we employed the variance inflation factor (VIF) to evaluate the presence of multicollinearity among the environmental factors. Variables with a VIF greater than 10 were excluded to mitigate the issue of multicollinearity. Consequently, we identified water temperature, dissolved oxygen, pH, conductivity, and flow velocity as the key environmental variables suitable for inclusion in the RDA. The biplots of RDA revealed that the first two principal axes accounted for 75.12% of the variance within the periphytic algae community (Figure 8). Within this context, water temperature, conductivity, and pH were identified as the environmental variables most significantly correlated with seasonal changes in community composition.

### 3.6 Relationship Between Landscape Pattern Indices and *β*‐Diversity of Periphytic Algae

**FIGURE 9:**
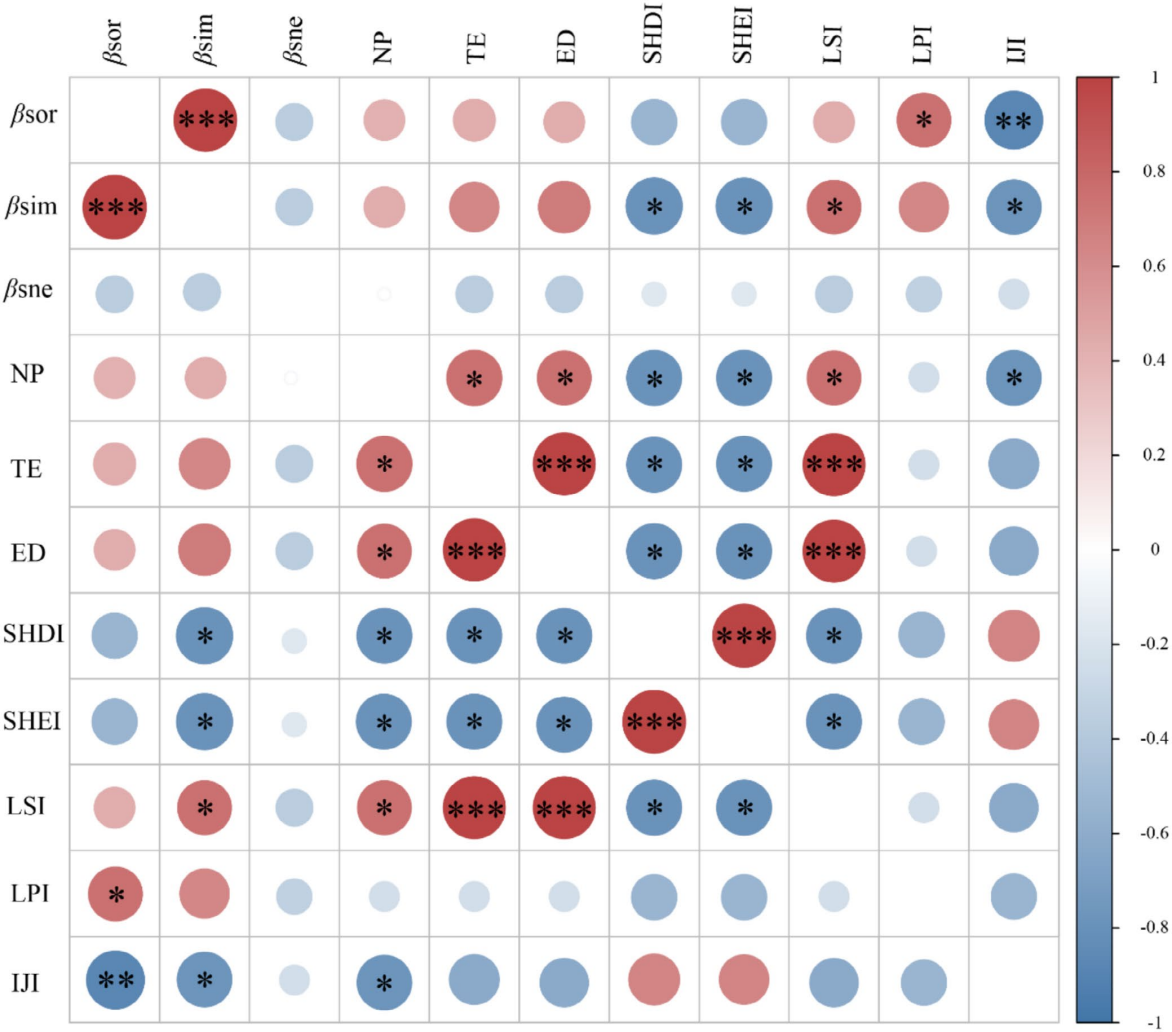
Spearman's rank correlation coefficients between *β*‐diversity of periphytic algae and landscape indices in the Chishui River. Red indicates a positive Spearman rank correlation coefficient, whereas blue signifies a negative Spearman rank correlation coefficient. *p < 0.05, **p < 0.01, ***p < 0.001. ED, edge density; IJI, the interspersion juxtaposition index; LPI, the largest patch index; LSI, the landscape shape index; NP, number of patches; SHDI, the Shannon diversity index; SHEI, the Shannon evenness index; TE, total edge; *β*sim, the turnover component; *β*sne, the nestedness component; *β*sor, the total *β*‐diversity.

Spearman's rank correlation coefficient, as presented in Figure 9, disclosed significant positive correlations between landscape fragmentation indices—namely, TE and ED—and the turnover component of *β*‐diversity. Concurrently, landscape heterogeneity indices, including the SHDI and SHEI, exhibited positive associations with the turnover components. Regarding landscape complexity metrics, the LSI demonstrated a positive correlation with the turnover component of *β*‐diversity, whereas the IJI exhibited a negative correlation. These findings reflect the distinct aspects of landscape complexity. Furthermore, the overall *β*‐diversity positively correlated with the LPI and negatively correlated with the IJI. Notably, the nestedness component of *β*‐diversity did not display significant correlations with any landscape pattern indices.

**FIGURE 10:**
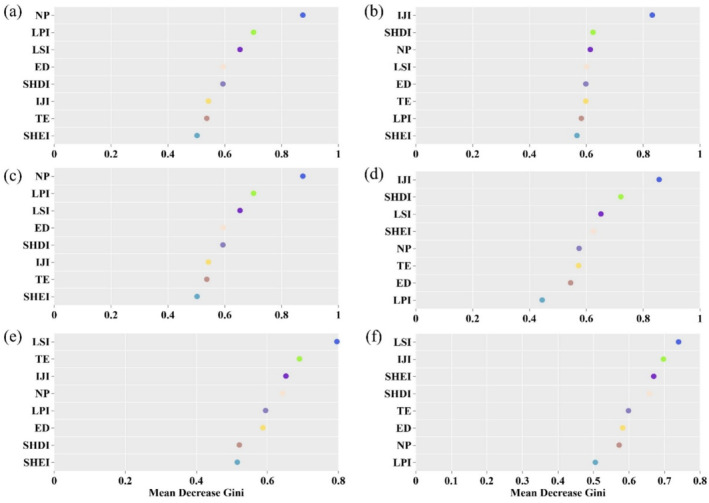
Importance ranking of landscape indices for *β*‐diversity of periphytic algae according to the random forest model. (a) total, (b) agriculture, (c) forestland, (d) grassland, (e) water bodies, (f) construction land. The mean decrease Gini value ascertains the significance of each landscape index within the model, with higher values denoting greater importance of the variable. ED, edge density; IJI, the interspersion juxtaposition index; LPI, the largest patch index; LSI, the landscape shape index; NP, number of patches; SHDI, the Shannon diversity index; SHEI, the Shannon evenness index; TE, total edge.

The random forest model analysis (Figure 10) revealed that the landscape fragmentation index, particularly the NP, exerted the most substantial influence on *β*‐diversity across various land use types and within forestlands. This effect was closely trailed by landscape complexity indices, such as the LSI and the LPI. In the context of agricultural and grassland areas, landscape complexity indices, particularly the IJI, were found to be the most influential on *β*‐diversity. Conversely, for water bodies and construction land, the LSI emerged as a particularly significant predictor. In summary, landscape fragmentation and complexity emerge as two pivotal determinants of *β*‐diversity among periphytic algae communities.

## 4 Discussion

### 4.1 Seasonal Variation of Periphytic Algae Community

Seasonal variations in mountain rivers lead to pronounced fluctuations in water levels and flow rates, which in turn create a mosaic of habitat heterogeneity (Sun et al. 2024). The diversity in spatial and temporal dimensions substantially shapes the composition and structure of periphytic algal communities. In this study, we undertook a comprehensive taxonomic examination of periphytic algae, resulting in the classification of 193 distinct taxonomic units across 8 phyla and 85 genera. Notably, the Bacillariophyta was predominant, representing 53.9% of the total species count. This dominance highlights the ecological significance of diatoms within the periphytic algae community. Our research in the Chishui River revealed that a mean periphytic algae density across dry, wet, and normal seasons was 1.43 × 10^5^ ind./cm^2^. Among the identified taxa, the Bacillariophyta exhibited the highest density, constituting the predominant component of the periphytic algae community. This prevalence of diatoms aligns with observations from ecological studies in adjacent aquatic systems (Hu et al. 2022; Shang et al. 2024). Prior studies have demonstrated that seasonal fluctuations exert a substantial influence on the composition of periphytic algae communities (Sofi et al. 2023). Our findings corroborate these observations, indicating a consistent pattern across different study sites. This concordance not only reinforces the validity of our study but also highlights the robustness of the ecological principles governing algal community dynamics. Utilizing one‐way analysis of variance (ANOVA), we revealed pronounced seasonal variations in the densities of the periphytic algae community within the Chishui River (*p* < 0.001). Notably, algal densities were substantially higher during the dry season compared to other seasons. Previous research has demonstrated that the dry season frequently results in elevated concentrations of nutrient salts in river systems (Várbíró et al. 2020). Furthermore, the Chishui River, in its natural state, is susceptible to seasonal flooding, a condition exacerbated by the absence of dam regulation. This hydrological phenomenon, particularly pronounced during the wet season, results in an increased river flow that intensifies the scouring of the riverbed substrate (Liu, Zhang, et al. 2021). Consequently, this process may contribute to a decrease in the density of periphytic algae species. In summary, these hydrological factors can partially account for the observed pattern in the Chishui River, where the density of periphytic algae reaches its maximum during the dry season and experiences a marked decline during the wet season.

In this study, we observed that the diversity of the periphytic algae communities within the Chishui River exhibited significant fluctuations in response to seasonal changes. Principal coordinate analysis (PCoA) of these communities revealed significant seasonal variations in *β*‐diversity, as depicted in Figure 6a. Subsequent analysis of similarities (ANOSIM) confirmed that these differences were statistically significant (*R* = 0.764, *p* = 0.001). Figure 6b illustrates that the *β*‐diversity of the periphytic algae community in the Chishui River was highest in the wet season, exhibiting significant differences compared to other seasons. *β*‐diversity, in contrast to *α*‐diversity and *γ*‐diversity, specifically highlights the variation in community composition across spatial and temporal gradients (Chen et al. 2020). Variations in habitat heterogeneity across seasons influence the biodiversity of periphytic algae communities (Tan et al. 2023). A decrease in habitat heterogeneity typically results in community homogenization (Han et al. 2024). Consequently, for periphytic algae, increased habitat heterogeneity is inversely correlated with the degree of community homogenization.

A pronounced decrease in algal community *β*‐diversity was observed during the dry and normal seasons as opposed to the rainy season. This observation is partly attributed to the elevated sediment load in these seasons. These results likely responsible for the reduction in habitat heterogeneity at the sampling sites within the study area. Concurrently, the augmentation of sediment volume during the dry and normal seasons exerts additional influences on the algal community (Chang et al. 2022). This phenomenon could plausibly account for the heightened homogenization observed within algal communities during these periods. Therefore, to preserve the integrity of the Chishui River's ecosystem, it is imperative to maintain habitat diversity, which is crucial for fostering and sustaining the biodiversity of the river.

### 4.2 The Correlation Between Environmental Drivers and Periphytic Algal Communities

The hydrological characteristics of aquatic ecosystems are pivotal in mediating energy transfer and material cycling, serving as a fundamental component for the conservation of biodiversity (Mulik, Sukumaran, and Srinivas 2020). The structural dynamics of algal communities are intricately associated with multiple environmental variables within aquatic ecosystems. Consequently, the precise identification and prioritization of key environmental drivers influencing the distribution of algal species are essential for the effective conservation of algal biodiversity (Song et al. 2020). RDA of the periphytic algae community with environmental variables (Figure 8) revealed that water temperature, conductivity, and pH are the predominant factors influencing the algae community structure in the Chishui River (*p* < 0.05). Prior research has demonstrated that river ecosystems are influenced by a multitude of natural variables, with water temperature emerging as a particularly influential factor (Han et al. 2024). Our RDA result corroborates this notion, underscoring the significance of water temperature in shaping algal community dynamics. Water temperature significantly influences the reproductive dynamics of algal species by modulating the respiration rate during photosynthesis and the efficacy of nutrient assimilation (Li et al. 2023). Crucially, algal species exhibit varying optimal water temperature requirements for growth. For instance, green algae and cyanobacteria tend to thrive at elevated temperatures, whereas diatoms are better adapted to cooler conditions (Shang et al. 2024). Notably, the relative abundance of periphytic algae significantly increased during the dry season, particularly under low‐temperature regimes, as evidenced in Table 2. This trend corroborates the phenomenon described above. Furthermore, in our study, the prevalence of cyanobacteria, which are known to prefer higher temperatures, exhibited a significant decline from the rainy season to the dry season. This observed change in community composition is also instrumental in explaining the observed significantly higher densities of periphytic algae during the dry season in contrast to the rainy season.

In mountainous river ecosystems, electrical conductivity emerges as a pivotal factor influencing the distribution patterns of periphytic algae (Peng et al. 2023). Our study's findings, which revealed a significant correlation between electrical conductivity and algal community composition, substantiate this assertion. Electrical conductivity, which is determined by the presence of free ions within the water column, provides an indirect assessment of the concentration of dissolved inorganic salts. Within an optimal range, an increase in conductivity is positively associated with enhanced diatom densities (Shang et al. 2024). Furthermore, fluctuations in conductivity can modulate the diversity of periphytic algae communities, thereby influencing their growth, developmental, and reproductive processes. Our study's outcomes align with these observations and provide additional validation for our conclusions.

In addition, pH is a critical factor that shapes the structure of periphytic algae community (Shang et al. 2024). It is a key determinant in the growth of these organisms, as it modulates the efficiency of their photosynthetic processes (Mirzahasanlou et al. 2020). A weakly alkaline aquatic environment facilitates the absorption of CO_2_ by algae and enhances photosynthetic activity (Jakobsen et al. 2015). Consequently, this enhancement leads to an increase in primary productivity. In contrast, algal growth is typically constrained in acidic aquatic environments. Notably, both biodiversity and algal abundance are observed to diminish significantly as the pH of the water body decreases (Ni et al. 2024). RDA results also revealed a pronounced relationship between pH levels and periphytic algae.

### 4.3 Relationship Between Landscape Pattern Indices and *β*‐Diversity of Periphytic Algae

Compositional analysis of *β*‐diversity serves as a pivotal indicator for assessing fluctuations of biodiversity in the ecosystem, playing an essential role in preserving the stability of river ecosystems (Wu et al. 2021). In this study, our analysis revealed that species turnover constituted the overwhelming majority (96.7%) of the total *β*‐diversity. This finding suggests that the turnover component is a more significant factor than species loss in accounting for the observed shifts in periphytic algae communities within our study area. The Chishui River basin encompasses a vast area, characterized by significant topographic variation and distinct climatic conditions. Collectively, these attributes drive the high turnover observed in the region (Heino and Tolonen 2017). Moreover, the diverse adaptive strategies of periphytic algae to their specific habitats may also be instrumental in driving community shifts, thereby generating elevated rates of species turnover across sites (Wu et al. 2021). Specifically, when nestedness component is the predominant component of *β*‐diversity, conservation efforts should prioritize areas with high biodiversity (Baselga 2010). Conversely, when the turnover component significantly contributes to total *β*‐diversity, a broader conservation approach that encompasses the entire watershed is warranted. Thus, a comprehensive conservation strategy for the study area is pivotal for preserving this region's biodiversity.

Investigating the relationship between landscape pattern indices and the *β*‐diversity of periphytic algae can enhance our comprehension of the mechanisms by which land‐use changes impact riverine ecosystems (Bomfim et al. 2023). Analysis of the random forest model revealed that, among numerous landscape indices, landscape fragmentation and complexity were the predominant factors shaping the *β*‐diversity of periphytic algae (Figure 10). Additionally, patch shape emerged as a significant landscape pattern indicator. Prior research has demonstrated that increased irregularity of patch edges and edge density can enhance the diversity of edge habitats, thereby fostering overall biodiversity (Fan et al. 2017). Our results align with prior observations in the field.

The impacts of landscape fragmentation on biodiversity are primarily manifested through alterations in the number of patches, which can subsequently influence species distribution and community composition (Rybicki, Abrego, and Ovaskainen 2020). Prior research has documented that increasing landscape fragmentation tends to coincide with an expansion in the number of patches, facilitating the coexistence of a greater diversity of species (Martello et al. 2023). Within the study area, an increase in patch number is likely to enhance both landscape connectivity and biodiversity (Herrero‐Jáuregui et al. 2022). Our research likewise observed a strong relationship between patch number and the *β*‐diversity of periphytic algae. This correlation highlights the significance of preserving an adequate number of habitat patches within the Chishui River basin to safeguard the biodiversity.

Notably, an increase in patch number within a landscape is often correlated with a reduction in individual patch sizes. These smaller habitat patches are critical for maintaining habitat connectivity (Wang et al. 2024). However, substantial decreases in patch area can adversely impact species richness (Jin et al. 2023). Therefore, striking a balance between patch number and size is essential for optimizing the ecosystem's capacity to sustain biodiversity.

## 5 Conclusion

This research has uncovered pronounced seasonal fluctuations in the community structure and diversity of periphytic algae within the Chishui River. Notably, environmental heterogeneity among sampling sites was reduced during the dry and normal seasons, corresponding to a marked decline in *β*‐diversity during these periods relative to the wet season. Thus, preserving environmental heterogeneity is essential for bolstering the biodiversity of the Chishui River ecosystem. Furthermore, these findings indicate that species turnover is the primary component driving *β*‐diversity. This suggests that comprehensive conservation efforts across the entire Chishui River basin are necessary to effectively conserve biodiversity.

In addition, this study also underscores the correlation between landscape pattern indices and the *β*‐diversity of periphytic algae. Ensuring an adequate number of habitat patches, along with a balanced distribution of patch sizes, is essential for sustaining the aquatic environment within the Chishui River basin.

In summary, this investigation into the relationship between the *β*‐diversity of periphytic algae and land use patterns offers novel insights and potential research directions for examining the *β*‐diversity of other aquatic organisms. Concurrently, this study on the periphytic algae in the Chishui River, a river characterized by natural flow, presents a valuable reference point for comparative analyses with other dammed rivers.

## Author Contributions

**Xiaopeng Tang:** conceptualization (lead), data curation (lead), methodology (lead), software (lead), writing – original draft (lead). **Haoyun He:** data curation (lead), software (lead). **Qiang Qin:** investigation (lead), validation (lead). **Fei Xu:** investigation (lead), validation (lead). **Fei Liu:** funding acquisition (lead), investigation (lead), validation (lead). **Fubin Zhang:** funding acquisition (lead), investigation (lead), writing – review and editing (lead).

## Conflicts of Interest

The authors declare no conflicts of interest.

## Data Availability

All data generated or analyzed in this study are contained in the Science Data Bank (https://www.scidb.cn/en/s/NvMbMr).
